# Association between physical activity and knee osteoarthritis: a comprehensive systematic review and meta-analysis

**DOI:** 10.7189/jogh.15.04173

**Published:** 2025-06-13

**Authors:** Xiaofeng Cui, Fangfang Xie, Jiahe Cui, Yukui Tian, Xue Bai, Lei Guo, Junchang Liu, Fei Yao

**Affiliations:** 1 The Fourth Clinical Medical College, Xinjiang Medical University, Urumqi, China; 2 School of Acupuncture-Moxibustion and Tuina, Shanghai University of Traditional Chinese Medicine, Shanghai, China; ^3^College of Traditional Chinese Medicine, Xinjiang Medical University, Urumqi, China ^4^Shanghai Municipal Hospital of Traditional Chinese Medicine, Shanghai University of Traditional Chinese Medicine, Shanghai, China

## Abstract

**Background:**

Knee osteoarthritis (KOA), a debilitating chronic degenerative joint disease, substantially compromises patients' functional capacity and quality of life. Although physical activity (PA) has been recognised as a modifiable risk factor in the development of KOA, its potential protective role remains debated. To clarify this clinical uncertainty, we conducted a rigorous systematic review and meta-analysis to comprehensively assess the association between PA levels and the risk of incident KOA.

**Methods:**

We systematically searched PubMed, EMBASE, Cochrane Library, CINAHL, Web of Science databases for published observational studies on the association between PA and knee osteoarthritis. Following the PRISMA guidelines, we selected English literature from inception to publication on 21 September 2024, and assessed study quality according to the Newcastle-Ottawa scale. Our protocol is available on PROSPERO.

**Results:**

Our systematic screening identified 14 eligible observational studies (13 cohort studies and one case-control study) involving 507 696 participants with 27 412 incident cases of KOA. A pooled analysis comparing levels of PA intensity showed a 26% increased risk of knee OA for high PA compared with moderate PA (relative risk (RR) = 1.26; 95% CI = 1.17–1.37). In contrast, neither high PA (RR = 1.02; 95% CI = 0.84–1.23) nor moderate PA (RR = 0.94; 95% CI = 0.84–1.05) showed protective effects compared with low PA reference groups. Stratified analyses showed nonsignificant associations in cohort studies (RR = 1.06; 95% CI = 0.87–1.29) compared with case-control studies (RR = 0.41; 95% CI = 0.20–0.83). Notably, regional subgroup analyses showed comparable PA-related risks between European (RR = 1.01; 95% CI = 0.74–1.38) and North American populations (RR = 1.03; 95% CI = 0.81–1.31). Crucially, gender-stratified analyses demonstrated no significant differential risk in males (RR = 1.20; 95% CI = 0.54–2.70) *vs*. females (RR = 0.73; 95% CI = 0.29–1.82).

**Conclusions:**

This systematic review comprehensively demonstrated a dose-dependent relationship between physical activity intensity and the risk of knee osteoarthritis. Our meta-analysis showed that high PA levels significantly increased the risk of knee osteoarthritis compared with moderate PA. In addition, limited evidence suggested that exceeding international PA guidelines may increase the risk of osteoarthritis (RR = 1.18; 95% CI = 1.02–1.35). However, future studies need to be executed to further define the type of activity, optimal dose and duration required to effectively reduce the risk of knee osteoarthritis.

**Registration:**

PROSPERO: CRD42024600175

Knee osteoarthritis (KOA), a prevalent chronic degenerative joint disease, is pathologically characterised by progressive degradation of articular cartilage. This structural deterioration triggers subchondral bone remodelling and synovial tissue changes, resulting in periarticular pain, oedema, and restricted range of motion. Functional impairment and persistent nociception remain the cardinal clinical manifestations that determine the severity of the disease [1,2]. Recent epidemiologic studies have estimated the 1-year prevalence of symptomatic knee osteoarthritis to be 13.8% among individuals aged ≥40 years, compared with a point prevalence ranging from 10.3 to 13.83% among adults aged ≥45 years in the global population. the prevalence is higher in women than in men [3,4]. As life expectancy increases and the global population ages, this trend is anticipated to persist. Middle-aged and older patients are likely to experience joint dysfunction and limited ambulation, which not only severely affects their quality of life and impairs their daily activities, but also has a significant economic impact on the health care system and social burden [5,6].

Currently, pharmacotherapy is an effective treatment modality for knee osteoarthritis. Among the available therapeutic options, intra-articular corticosteroid injections have emerged as a first-line intervention, demonstrating superior clinical efficacy compared with alternative pharmacological approaches [7,8]. However, clinicians should be cognizant of the potential adverse effects, including transient hyperglycaemia in diabetic patients, as well as the risks of vascular complications, hypersensitivity reactions, and ocular disorders associated with this treatment modality [9]. Neuromuscular training has been recognised as an effective non-pharmacological intervention for knee osteoarthritis. It has shown significant analgesic effects while enhancing joint functionality and skeletal muscle strength [10]. Consequently, there is an urgent need to develop cost-effective, feasible, and scalable prevention and management strategies for knee osteoarthritis.

A growing body of clinical evidence indicates that physical activity is a modifiable risk factor for KOA. However, whether physical activity confers protective or detrimental effects on KOA progression remains inconclusive. A meta-analysis found no significant association between overall physical activity and an increased risk of KOA [11]. Conversely, an observational study reported that male professional football players exhibited a 2.3-fold higher risk of symptomatic KOA (odds ratio (OR) = 2.25) and a 3.987-fold increased risk of radiographic KOA compared with the general male population [12]. In contrast, another study demonstrated a protective effect of exercise against KOA, with physically active individuals showing a 0.75-fold lower risk of developing KOA than their sedentary counterparts [13]. Current evidence suggests that moderate physical activity may exert a protective effect against KOA, while excessive exercise appears to increase the risk of KOA development. However, the optimal exercise intensity for KOA prevention remains unclear. Recent meta-analyses have demonstrated that physical activity enhances lower extremity muscle strength, which is particularly relevant to KOA pathogenesis. Evidence highlights a significant association between knee extensor weakness and incident KOA, suggesting that optimising quadriceps strength may represent a viable preventive strategy against KOA development [14]. To our knowledge, previous studies have primarily focused on evaluating the efficacy and safety of physical activity in relation to KOA, while few meta-analyses have specifically assessed the impact of physical activity intensity on KOA progression. Therefore, in this systematic review and meta-analysis, we evaluated whether the intensity of physical activity reduces the risk of knee osteoarthritis. We also examined equally important potential effect modifiers, including body weight, sex, and age. Although current evidence regarding the optimal duration and intensity of physical activity for KOA risk reduction remains limited, our findings may provide valuable insights for clinical guidelines and intervention strategies aimed at optimising primary prevention approaches for KOA.

## METHODS

This study was conducted in accordance with the PRISMA guidelines for reporting the results of systematic reviews. The study protocol, including the complete methodology of this synthesis, is available on https://www.crd.york.ac.uk/PROSPERO/#recordDetails (CRD42024600175).

### Literature search

We conducted a systematic search of PubMed, EMBASE, Cochrane Library, CINAHL, and Web of Science for English-language studies published from database inception to 21 September 2024. The search strategy employed Boolean operators to:

1) combine physical activity-related terms using OR operators, and

2) link these terms with KOA outcome descriptors using AND operators (**Online Supplementary Document**). This approach, which focused on human studies, was designed to optimise both sensitivity and specificity in identifying relevant publications that met our predefined inclusion criteria, thereby establishing a robust evidence base for our analysis.

### Inclusion and exclusion criteria

Two authors (XFC and FFX) independently screened all titles and abstracts according to predefined inclusion and exclusion criteria. Studies were eligible for inclusion if they:

1) investigated the association between PA and KOA;

2) utilised observational study designs (cohort or case-control studies);

3) reported ORs or relative risks (RRs); and

4) provided either these effect measures along with corresponding 95% confidence intervals (CIs) or sufficient data for their calculation.

We excluded:

1) studies with unavailable full texts;

2) studies lacking relevant outcome data;

3) non-original research (reviews, meta-analyses, letters, practice guidelines);

4) studies combining PA with other interventions; and

5) studies not aligned with our research objectives.

### Data extraction

Two reviewers independently assessed the eligibility of all identified studies, and relevant publications were selected for inclusion. Any discrepancies in study selection were resolved through consensus-based discussions. To examine the association between PA and KOA, we extracted the following data elements: first author surname, case numbers, year of publication, participant sex, sample size, study design and location, three-level PA classification, relative risk estimates with corresponding 95% CIs, and adjusted covariates. Our analysis specifically focused on three PA modalities:

1) vigorous PA,

2) quantitative PA (assessed by frequency or exact weekly bouts), and

3) lifetime PA.

The quantitative PA evaluation encompassed the frequency of PA or the precise number of PA cycles per week. Among the 14 studies selected, one reported relative risk estimates with moderate levels of PA as the reference group, and we converted the reported relative risk estimates (RRi) to reciprocal values [15]. The remaining studies directly involved low PA.

### Study quality

Two researchers (XFC and FFX) independently assessed the quality of the included studies using the Newcastle–Ottawa Scale (NOS). In cases where inconsistencies in study quality assessments arose, an additional experienced researcher was invited to resolve the discrepancies and determine the final quality rating. The NOS scale, with a maximum score of 9, was primarily used to evaluate case-control studies (exposure, selection, and comparability) and cohort studies (comparability, outcomes, and selection). Studies with a score of less than 4 were considered to be of low quality, while those with a score greater than 5 were identified as high-quality studies [16].

### Data analysis

The extracted ORs from case-control studies and RRs from cohort studies were interpreted as RRi to quantify the association between PA exposure and KOA development. When available, we directly extracted the most comprehensively adjusted RR estimates reported in the original studies. For studies where RR values and 95% CIs were not provided, we calculated these measures using Stata, version 15.1 (StataCorp, College Station, Texas, USA). We then calculated the natural logarithm of relative risk estimates (log(RRi)) and their corresponding standard errors (SEsi), where Sesi = (log(upper 95% CI limit of RR) - log(RR))/1.96. A random-effects model was exploited to account for the weighted averages of log(RRi)s while considering the heterogeneity of effect measures. Furthermore, log (RRi)s were weighted with wi = 1/(si^2^ + t^2^), where si represented the standard error of log (RRi) and t^2^ denoted the restricted maximum likelihood estimate of the total variance Q- and *I*^2^-statistics were used to determine heterogeneity [17]. We utilised *I*^2^ statistics to quantify the between-study heterogeneity, as *I*^2^<25% were commonly acknowledged as a key indicator of low heterogeneity [17]. To assess potential publication bias, we employed three complementary approaches:

1) visual inspection of funnel plot symmetry,

2) Begg's rank correlation test [18], and

3) Egger's linear regression method [19].

We performed subgroup analyses to investigate the association between PA and KOA, stratified by:

1) sex (male, female),

2) study design (case-control, cohort),

3) PA intensity levels (high, moderate, low),

4) number of adjustment factors considered (age, BMI, education level), and

5) geographic region (North America, Europe). These analyses took into account potential effect modification by these key covariates.

Statistical analyses were conducted with Stata, version 15.1 (StataCorp, College Station, Texas, USA), and relative risk estimates were reported with corresponding 95% CIs. The statistical significance level for this study was set at α = 0.05.

## RESULTS

A total of 507 696 subjects and 27 412 cases were included in this meta-analysis. We initially conducted a systematic search across five databases, identifying 25 737 studies (3967 from PubMed, 5801 from EMBASE, 4210 from the Cochrane Library, 2281 from CINAHL, and 9478 from Web of Science). After removing duplicate entries, 14 766 articles remained for further evaluation. Following the exclusion of studies deemed unrelated based on their titles and abstracts, we conducted an in-depth review of 86 studies. Of these reviewed studies, 65 were excluded due to the inability to extract PA or relative risk data; an additional seven were excluded because they were written in a non-English language. Consequently, a total of 14 studies were retained for analysis, providing 16 distinct RR estimates (Table S1 in the **Online Supplementary Document**).

Table S1 in the **Online Supplementary Document** shows the key characteristics of PA and KOA in 13 cohort studies [15,20–31] and one case-control study [32]. These studies were conducted in five countries: the Netherlands [20], the England [15,22], the Finland [27,32], the Canada [24],and the USA [21,23,25,26,28–31]. Among the analysed factors, 56% were adjusted for age and obesity, while 44% of the risk estimates were adjusted for additional factors such as trauma, race, and education level. Additionally, eight of the included studies [21,22,24,26,28–31] classified PA as follows: high PA for over 150 minutes per week, low PA for less than 30 minutes, and moderate PA for 30 to 150 minutes.

### Subgroup analyses

#### Comparison of high and low PA levels

The results of the random-effects model demonstrated that there was no statistically significant association between high levels of physical activity and a reduced risk of knee osteoarthritis when compared with low levels of physical activity (RR = 1.02; 95% CI = 0.84–1.23). Furthermore, the model identified substantial heterogeneity among the studies (*I*^2^ = 98.9%; *P* < 0.001) (Figure S2 in the **Online Supplementary Document**).

#### Sex

When the data were stratified by gender, we observed that men exhibited a stronger association between physical activity and knee osteoarthritis (RR = 1.20; 95% CI = 0.54–2.70) compared with women (RR = 0.73; 95% CI = 0.29–1.82). However, the overlapping confidence intervals indicate that this difference did not achieve statistical significance (Figure S3 in the **Online Supplementary Document**).

#### Geographical regions

Our analysis stratified by geographical region indicated that PA was associated with a similar risk of knee osteoarthritis in both the European population (RR = 1.01; 95% CI = 0.74–1.38) and the North American population (RR = 1.03; 95% CI = 0.81–1.31) (Figure S4 in the **Online Supplementary Document****)**.

#### Type of design

When the analysis was stratified by study design type, a 6% increase in the risk of knee osteoarthritis was observed after excluding case-control studies (RR = 1.06; 95% CI = 0.87–1.29). In addition, the pooled estimates from both case-control and cohort studies indicated a 2% increased risk of knee osteoarthritis (RR = 1.02; 95% CI = 0.84–1.23) (Figure S5 in the **Online Supplementary Document**).

#### Publication bias

Analyses of publication bias were performed utilising a funnel plot, Begg’s rank correlation test (*P* = 0.56), and Egger’s linear regression test (*P* = 0.88). None of these methods indicated the presence of significant publication bias (*P* > 0.05) (Figure S6–8 in the **Online Supplementary Document**).

### KOA incidence by PA level

We performed a meta-analysis to evaluate the effects of low, moderate, and high levels of PA on the risk of knee osteoarthritis across 12 studies, encompassing a total of 14 risk estimates. The results demonstrated that, compared with low PA levels, moderate PA levels were not significantly associated with changes in the risk of knee osteoarthritis (RR = 0.94; 95% CI = 0.84–1.05). In contrast, high PA levels were associated with a 26% increased risk of knee osteoarthritis compared with moderate PA levels (RR = 1.26; 95% CI = 1.17–1.37). Additionally, based on limited evidence and consistent with international guidelines, high PA levels may be linked to an elevated risk of knee osteoarthritis (RR = 1.18; 95% CI = 1.02–1.35) (Figure S9–11 in the **Online Supplementary Document**).

### Study quality

The quality assessment of the studies is summarised (Table S2 in the **Online Supplementary Document**). Due to the non-normal distribution of the Newcastle-Ottawa Scale (NOS) scores (Shapiro-Wilk test, *P* < 0.05), study quality was characterised using medians and interquartile ranges. The NOS scores for the included studies ranged from a minimum of 5 to a maximum of 7, with a median of 6.5 (IQR = 6–7), reflecting high overall methodological quality. The included studies accounted for a wide range of potential confounders, such as age, sex, and body mass index. All included studies met a predetermined threshold of high quality criteria (NOS≥5). To minimise bias, sensitivity analyses then included all eligible studies (NOS≥5, n = 14). Based on the questionnaire assessments, these studies exhibited satisfactory methodological quality.

## DISCUSSION

The pathogenesis of KOA, a degenerative joint disease, is intricately associated with the aging process and levels of physical activity. Extensive epidemiological evidence has consistently demonstrated a significant increase in the prevalence of KOA with advancing age. This trend is primarily attributed to age-related degenerative changes in articular cartilage, characterised by a diminished repair capacity of chondrocytes, which subsequently impairs the synthesis of collagen and proteoglycans within the cartilage matrix [33,34]. This decline in matrix composition results in compromised tolerance of cartilage to mechanical stress. Sedentary behaviour or physical inactivity represents a substantial risk factor for the progression of KOA. The long-term lack of physical activity leads to muscle atrophy, weakening the muscles surrounding joints due to insufficient exercise. This reduction diminishes both support and protection for joints while decreasing overall joint stability, thereby further increasing the risk of articular cartilage wear [35]. Conversely, regular physical activity has been shown to enhance skeletal muscle mass and strength in the lower limbs, improve dynamic stability at the knee joint, and reduce mechanical damage to cartilage caused by abnormal stress. In elderly individuals, consistent engagement in physical activity promotes synovial fluid circulation, enhances nutritional supply to articular cartilage, and improves metabolic rates as well as repair capacities within this tissue. Such effects can be likened to those observed in younger individuals' physiological functions regarding their joints; thus potentially contributing to an older individual's physiological age being closer to that of a younger person [36]. Concurrently, participation in physical activities has been shown to bolster muscle strength, augment skeletal muscle mass in lower limbs, improve joint stability, reduce risks associated with joint injuries – ultimately mitigating potential disability outcomes [37].

As a crucial tool for prevention and treatment, clinical studies have demonstrated that PA can effectively alleviate the clinical symptoms of patients with knee osteoarthritis, enhance joint function, and significantly improve quality of life [38],PA yields benefits through a multidimensional mechanism:

1) it increases the strength of the quadriceps and other core muscles, enhances skeletal muscle quality, and optimises stress distribution across the joints;

2) it promotes synovial fluid metabolism, modulates levels of various inflammatory factors, and mitigates inflammatory responses within the joints.

Clinical research has confirmed that regular aerobic exercise can substantially reduce joint pain, enhance joint mobility, and postpone the necessity for joint replacement [39,40]. The World Health Organization (WHO) guidelines on physical activity and sedentary behaviour recommend that healthy adults engage in daily activities at an intensity level comprising:

1) at least 150–300 minutes per week of moderate-intensity aerobic physical activity; and

2) at least 75–150 minutes per week of vigorous-intensity aerobic physical activity [41]. Consequently, exercise intervention serves not only as a means for symptom management but also as a vital strategy for promoting healthy aging.

Therefore, we conducted a random-effects meta-analysis based on the available evidence suggesting that the relationship between different intensities of exercise interventions and the risk of KOA development presents a complex dose-response profile. The current study demonstrated that the relative risk ratio for KOA development did not reach statistical significance when comparing high PA to low PA (RR = 1.02; 95% CI = 0.84–1.23). This finding suggests a potential nonlinear association between exercise intensity and KOA risk, which aligns with results from previous studies [42]. Furthermore, high PA was associated with a 26% increase in the risk of knee osteoarthritis compared with moderate PA (RR = 1.26; 95% CI = 1.17–1.37). In accordance with international guidelines, high PA increased the risk of knee osteoarthritis by 18% (RR = 1.18; 95% CI = 1.02–1.35), consistent with earlier clinical investigations [42,43].Additionally, moderate PA did not significantly reduce the risk of knee osteoarthritis when compared to low PA (RR = 0.94;95% CI = 0.84–1.05),which contradicts findings from prior research [44,45]. This discrepancy may be attributed to the fact that included literature primarily focused on extracting data related to exposure factors such as PA without explicitly reporting its dose intensity, potentially introducing bias into our results. Based on the above content, the nonlinear relationship between activity intensity and the risk of KOA indicates the existence of a potential threshold effect. Although moderate PA can provide a protective balance between joint load and muscle strengthening, exceeding this threshold (high PA) may increase mechanical stress on the cartilage. Moderate PA is an effective means of preventing knee osteoarthritis, while low or high PA does not bring significant benefits, which is consistent with a previous study [46].

### Subgroup analysis of KOA

The analysis stratified by study type and region indicated that the findings of physical activity studies on knee osteoarthritis were consistent across different categories, with no significant influence from either the type of study or geographical region. However, some potential bias may arise due to the predominance of literature sourced from Europe and North America. In contrast to certain previous studies, our subgroup analyses revealed no significant sex differences in the association between PA and KOA risk [47].This can primarily be attributed to several factors: the age distribution of women in the included samples was predominantly within the premenopausal range, a period characterised by elevated oestrogen levels that help maintain protective effects on bone and joint health. It is well established that oestrogen plays a crucial role in maintaining bone and joint integrity. Previous research has demonstrated that oestrogen reduces endoplasmic reticulum stress and stress-induced apoptosis in articular chondrocytes, thereby mitigating critical events conducive to cartilage degradation associated with osteoarthritis [48]. Conversely, postmenopausal women experience an accelerated loss of osteocalcin leading to abnormalities in cartilage metabolism as well as anatomical stresses resulting from a sudden decline in oestrogen levels. Consequently, their risk for developing knee OA is significantly higher than that observed in men; however, this risk appears substantially reduced through oestrogen supplementation [48].Given these considerations alongside the reviewed literature indicating that participants were predominantly premenopausal women, it follows that there would be minimal differences between sexes regarding the prevalence of knee osteoarthritis.

### Biological mechanisms

Based on our current understanding, several mechanisms can be elucidated: knee osteoarthritis is not confined to damage of the articular cartilage; rather, it represents a disease that encompasses the entire knee joint, including subchondral bone, ligaments, synovium, and skeletal muscle. Damage to the articular cartilage is typically instigated by normal wear and tear, abnormal mechanical loading, external injury, and aging [49–51]. According to existing research evidence, the effects of high-intensity physical activity on joint degeneration can be summarised through the following mechanisms: high-intensity physical activity significantly elevates the mechanical load on articular cartilage [52]. This sustained load not only directly accelerates metabolic degradation of the cartilage matrix but also exacerbates cartilage degeneration by triggering pro-inflammatory factor release from inflammatory cells – thereby creating a vicious cycle [37]. Furthermore, elevated loads influence the remodelling processes of subchondral bone. Uncoupled remodelling of subchondral bone emerges as a critical factor contributing to an imbalanced distribution of mechanical forces within the knee joint, which further compromises both subchondral and superficial cartilage integrity [53]. Additionally, according to the ‘window effect’ theory regarding cartilage metabolism, intermittent exercise promotes chondrocyte proliferation while high-intensity exercise induces apoptosis. This distinction elucidates why high-intensity exercise may accelerate degenerative processes whereas low-intensity exercise might fail to provide adequate metabolic stimulation.

National and international studies have confirmed that patients with knee osteoarthritis exhibit significant mechanical abnormalities in the periprosthetic muscle groups. These abnormalities are primarily characterised by a reduction in the cross-sectional area of the quadriceps muscle, a transformation of muscle fibre types towards fast-twitch fibres, and a decrease in peak knee extension torque. Notably, the decline in quadriceps muscle strength is identified as the core pathological factor [54,55]. Additionally, muscular imbalances and synergistic dysfunctions within the dynamic stabilising structures of the knee joint – specifically, the quadriceps-popliteus system – are evident. Abnormal ratios of popliteus to quadriceps peak torque significantly elevate the risk of dynamic joint instability [56]. Conversely, sedentary behaviour and physical inactivity contribute to the progression of osteoarthritic knee disease through dual pathways: first, they impair synovial fluid circulation and lead to skeletal muscle atrophy, which diminishes joint stability; second, they may accelerate biological aging processes and heighten disability risks [35].Clinical studies indicate that moderate-intensity physical activity operates via a dual mechanism: it enhances quadriceps muscle strength while maintaining knee joint adduction moments within safe thresholds. This approach demonstrates broad clinical efficacy and facilitates cartilage loading within physiologically adaptive ranges when compared with low-intensity exercise. Thus, it suggests that the synergistic effects of recovering muscle strength alongside load regulation represent key pathways for improving KOA outcomes.

### Strengths and limitations

The primary strength of this study lies in the effective integration of multiple research findings. Throughout the course of our investigation, we conducted detailed subgroup analyses that accounted for gender differences, regional characteristics, and the robustness of international guideline recommendations. Importantly, the literature included in this analysis has significantly enhanced the comprehensiveness, accuracy, and reliability of our research outcomes by adjusting for variables such as gender, age, trauma history, and body mass index. This provides a solid scientific foundation for further research and informed decision-making in related fields.

Regarding limitations, it is important to note that some studies included in our analysis did not account for recreational and occupational physical activities. This omission restricts a more comprehensive examination of the relationship between PA and the risk of knee osteoarthritis.

### Implications

Our findings show an association between physical activity and the risk of knee osteoarthritis, and provide guidance for patients on how to prevent the disease through physical activity. Despite the current research in this area, further research is needed to determine the optimal dose, intensity, and duration of PA to reduce the risk of knee osteoarthritis. Follow-up prospective studies should comprehensively address all aspects and levels of PA and systematically investigate the effects of PA on the severity of knee osteoarthritis. During this time, all confounding factors that could bias the results must be taken into account.

## CONCLUSIONS

Our comprehensive meta-analysis supports that high PA significantly increases the risk of knee osteoarthritis compared with moderate PA. Limited evidence suggested that high PA may increase the risk of knee osteoarthritis according to international guidelines (RR = 1.18; 95% CI = 1.02–1.35). However, future studies need to further define the type, optimal dose and duration of physical activity required to effectively reduce the risk of knee osteoarthritis.

## Additional material


Online Supplementary Document
