# Supplementary material for: Association between physical activity and knee osteoarthritis: a comprehensive systematic review and meta-analysis
Source: J Glob Health. 2025 Jun 13;15:04173. doi: 10.7189/jogh.15.04173 (PMC12163862; doi:10.7189/jogh.15.04173)
Supplement: Online Supplementary Document [file jogh-15-04173-s001.pdf]

Supplement to: Cui X, Xie F, Cui J, Tian Y, Bai X, Guo L, Liu J, Yao F. Association between physical activity and knee osteoarthritis: a comprehensive systematic review and meta analysis. J Glob Health. 2025;15:04173.

Physical activity and constipation: A systematic review of cohort studies
1. Full search terms
2. Characteristics of the 14 studies on physical activity and constipation risk (Table 1)
3. Quality of studies according to Newcastle-Ottawa Scale (Table 2)
4. Figures

Full search strategy

The full search syntax, represented by the pubmed database, is shown below:
(("Exercise"[Mesh]) OR (((((((((((((((((((((((((Exercises[Title/Abstract]) OR (Exercise, Physical[Title/Abstract])) OR (Exercises, Physical[Title/Abstract])) OR (Physical Exercise[Title/Abstract])) OR (Physical Exercises[Title/Abstract])) OR (Physical Activity[Title/Abstract])) OR (Activities, Physical[Title/Abstract])) OR (Activity, Physical[Title/Abstract])) OR (Physical Activities[Title/Abstract])) OR (Exercise, Aerobic[Title/Abstract])) OR (Aerobic Exercise[Title/Abstract])) OR (Aerobic Exercises[Title/Abstract])) OR (Exercises, Aerobic[Title/Abstract])) OR (Exercise, Isometric[Title/Abstract])) OR (Exercises, Isometric[Title/Abstract])) OR (Isometric Exercises[Title/Abstract])) OR (Isometric Exercise[Title/Abstract])) OR (Acute Exercise[Title/Abstract])) OR (Acute Exercises[Title/Abstract])) OR (Exercise, Acute[Title/Abstract])) OR (Exercises, Acute[Title/Abstract])) OR (Exercise Training[Title/Abstract])) OR (Exercise Trainings[Title/Abstract])) OR (Training, Exercise[Title/Abstract])) OR (Trainings, Exercise[Title/Abstract]))) AND (("Osteoarthritis, Knee"[Mesh]) OR ((((Knee Osteoarthritides[Title/Abstract]) OR (Knee Osteoarthritis[Title/Abstract])) OR (Osteoarthritis of the Knee[Title/Abstract])) OR (Osteoarthritis of Knee[Title/Abstract])))

Table S1. Characteristics of the 14studies on physical activity and knee osteoarthritis risk

	Gender	Region	Subjects	Cases	Relative Risk(95%CI) for high vs low PA	Relative Risk (95%CI) for high vs moderate PA	Relative Risk (95%CI) for moderate vs low PA	Low PA defned by	Moderate PA defned by	High PA defned by	Adjustment factors
Cohort studies											
Szilagyi al 2022	female	Netherlands	6084	713	1.17 (0.92 ,1.47)	1.17 （1.16,1.17）	1.00 (0.79 ,1.26）	Low	Moderate	High	age,months to allow-up, sub-cohort and BMI
Szilagyi al 2022	male	Netherlands	4874	351	1.85 (1.34 ,2.56)	1.46 （1.42,1.49）	1.27 (0.90 ,1.80)	Low	Moderate	High	age, months to follow-up, sub-cohort and BMI
Felson et al 2013	female and male	The United States	3,498	53	0.6 (0.3, 1.3)	None	None	PASE Lower 75%	None	High (upper 25%)	age, sex, BMI, WOMAC pain and KL grade and study of origin.
Zhu et al 2022	female and male	England	436,166	21119	1.14 (1.05,1.23)	1.14 （1.13,1.14）	1.00 (0.93,1.08)	Low （<1st tertile)	Moderate (1st-2nd tertile)	High (⩾ 2nd tertile)	gender, race or ethnicity, body mass index, smoking status, drinking status, annual household income, educational attainment, history of joint injury, and Townsend deprivation

											index
Cheng et al 2000	female and male	The United States	16,961	601	1.45 （0.57,1.53）	1.45 （1.22,1.92）	1（0.8,1）	Low (＜10)	Moderate (10－20)	High （>20)	None
Plotnikoff et al 2015	female and male	Canada.	4733	497	0.67 (0.41,1.11)	None	None	Weekly leisure activity (score ≤ 23, %)	None	Weekly leisure activity (score ≥ 24, %)	None
Perry et al 2020	female and male	The United States	908	209	1.98 (1.03 ,3.82)	1.32 （1.16,1.52）	1.5 (0.89 ,2.52)	Sedentary	Light Manual	heavy manual	age, BMI, sex, race & ethnicity.
Qin et al 2002	female and male	The United States.	694	56	1.52 (0.68,3.40)	1.97 （1.74,2.28）	0.77 (0.39,1.49)	Inactive （<10 min • wk）	Low （10-<150 min • wk）	Active（≥ 150 min • wk）	age, sex, BMI, and prior knee injury
Toivanen, et al 2009	female and male	Finland	823	94	0.5 (0.3,1.0)	0.63 （0.6,0.77）	0.8 (0.5,1.3)	Little	Irregular	Regular	all covariates,
Barbour et al 2003	female and male	The United States.	2573	251	1.33 (1.29,1.36)	1.22 （1.18,1.27）	1.08 (1.07,1.1)	Low (10－<150 min/week)	Medium (150－<300 min/week)	High (≥ 300 min/week)	age, sex, race, BMI, education, occupational activity, and prior knee injury
McAlindon et al 1999	female and male	The United States.	940	103	5.3 (1.2,24)	2.52 （1.71,3.58)	2.1 (0.7,6.7)	Low (0 Hours per Day)	Middle (1 to 2Hours per Day)	High (≥ 3Hours per Day)	age, sex, body mass index, weight loss, knee injury, health status, current smoking, and total caloric intake.
MD MSc et al	female and male	The United States.	2,819	77	1.90 (1.03,3.52)	1.71 （1.69,1.72）	1.11 (0.60,2.08)	Level 1	Level 2	Level 3 and 4	age，sex, knee injury, BMI , the remaining 11

2022											domains of the PASE, kneeling activities, squatting activities, in-and-out of squatting activities, and education.
Huang et al 2024	female and male	The United States	22,901	2705	1.08 (1.05,1.11)	1.29 （1.29,1.3）	0.84 (0.81,0.86)	Physical activity levels （1.4-17.1 MET-min' week)	Physical activity levels (17.7-42.9 MET-min' week)	Physical activity levels (145-1080 MET-min' week)	age, gender, race, level of education, BMI, smoking status, alcohol consumption, hypertension, diabetes, blood urea nitrogen, total calcium, phosphorus, triglycerides, uric acid, total cholesterol.
Martin et al 2013	male	England	1438	109	0.81 （0.79,0.83）	1.44 (0.71,2.88)	0.56 （0.29,1.11）	Inactive	Less active	Most active	income, childhood social class, family history of arthritis, history of knee injury and health status at 53y
Martin et al 2013	Female	England	1479	193	0.46 （0.43,0.49）	0.67 (0.37,1.21)	0.68 （0.40,1.15）	Inactive	Less active	Most active	income, childhood social class, family history of arthritis, history of knee injury and health status a
Case-control studies											
Manninen et al 2002	female and male	Finland	805	281	0.41 (0.20,0.81)	0.69 （0.53,0.89）	0.59 (0.38,0.91)	None regularly	Low	High	Body mass index,Knee injury,occupational

											exposures,

Table S2. Quality of studies according to Newcastle-Ottawa Scale

First author,Year, country	Selection (Max,score4)	Comparability (Max,score2)	Exposure(case-control) or outcome (cohort) (max,score3)
Cohort			
Szilagyi al 2022	3	2	2
Felson et al 2013	3	1	2
Zhu et al 2022	3	2	2
Cheng et al 2000	2	1	2
Plotnikoff et al 2015	3	1	2
Perry et al 2020	3	1	2
Qin et al 2002	3	1	3
Toivanen et al 2009	3	1	3
Barbour et al 2003	3	1	2
McAlindon et al 1999	3	1	3
MD MSc et al 2022	3	1	2
Huang et al 2024	3	2	2
Martin et al 2013	3	1	3
Case-control			
Manninenet al 2002	3	1	2

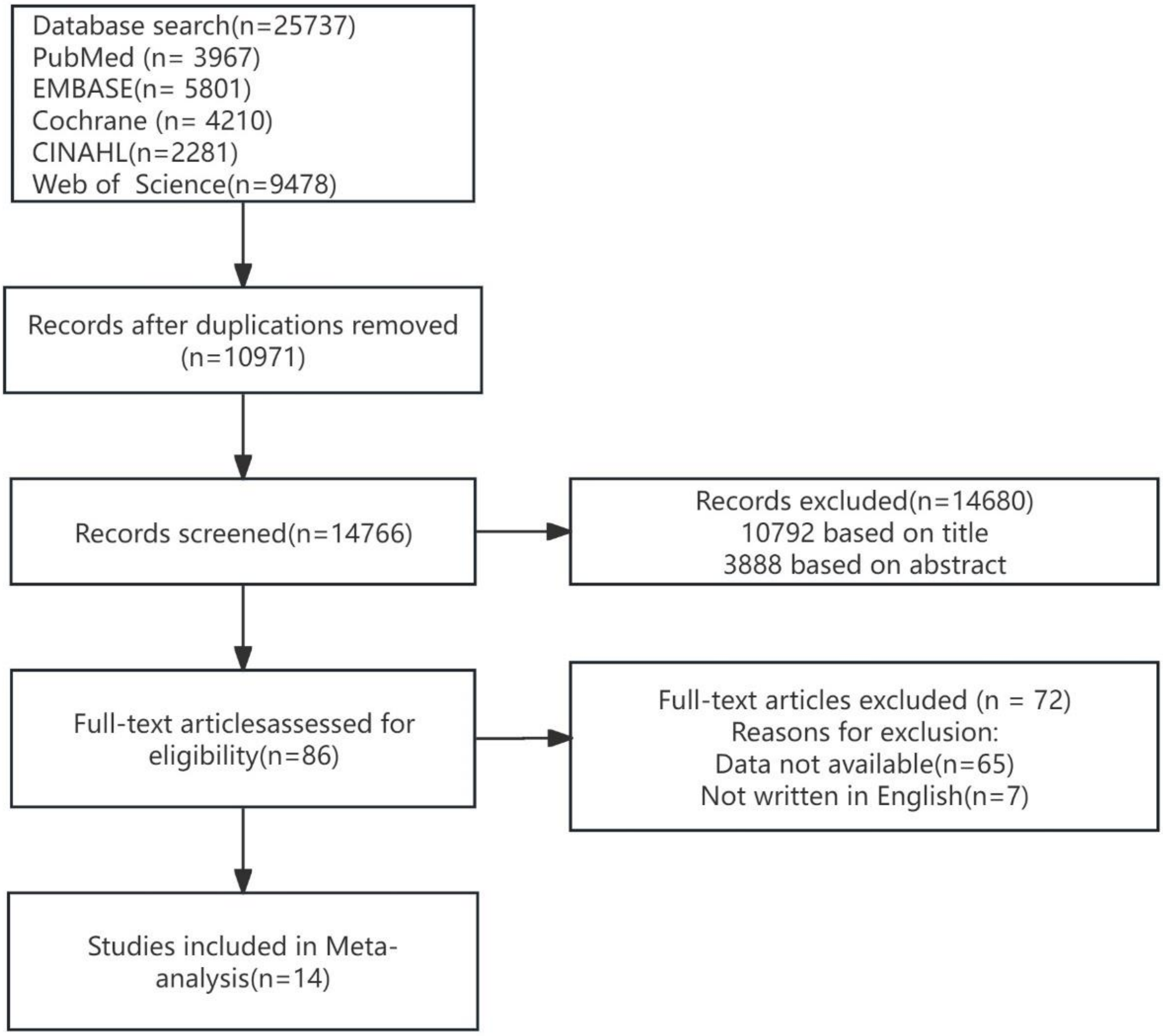

Figure S1. PRISMA fow diagram of identifcation and selection of eligible studies

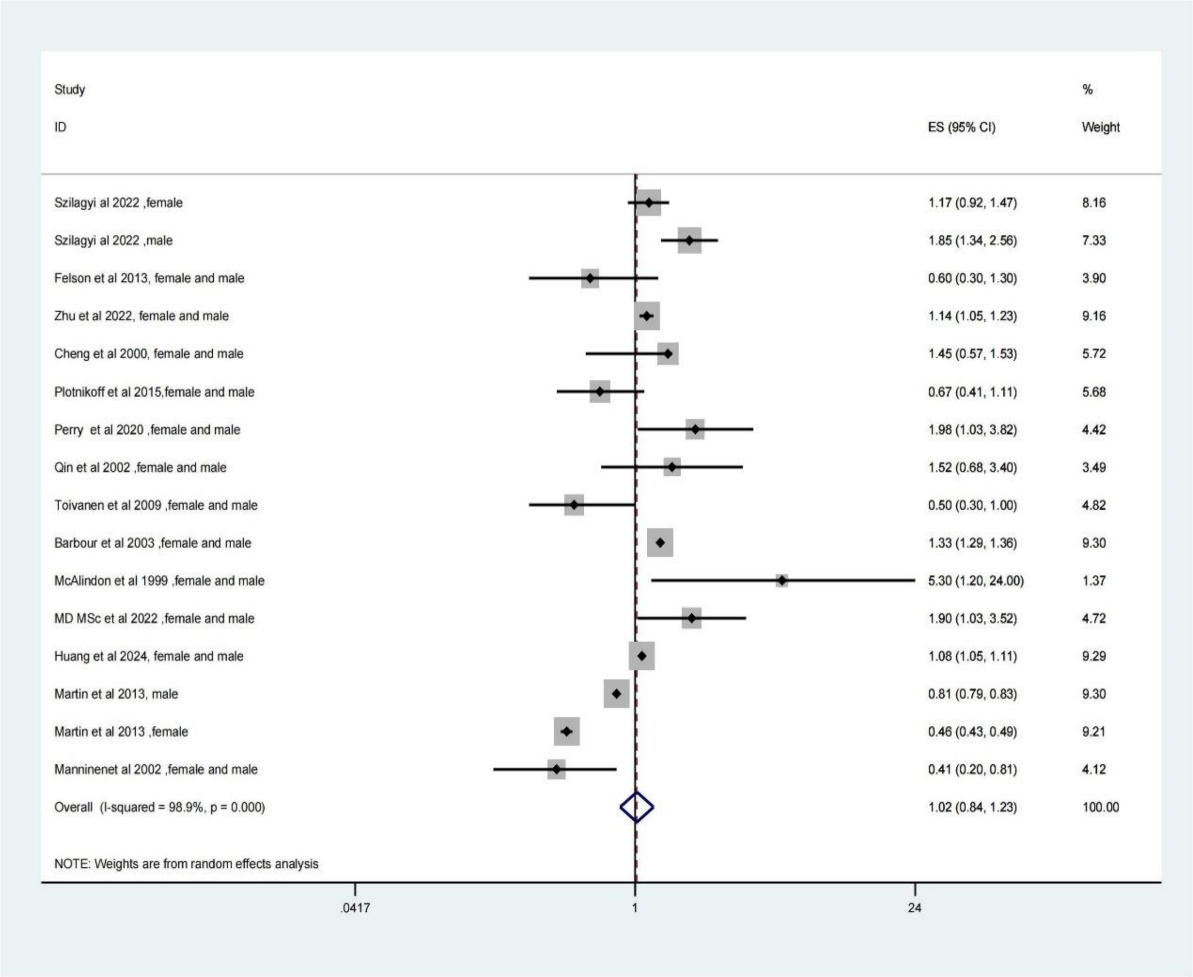

Figure S2. Forest plot of a random effects meta-analysis including 16 risk estimates of knee osteoarthritis for a high versus low level of PA

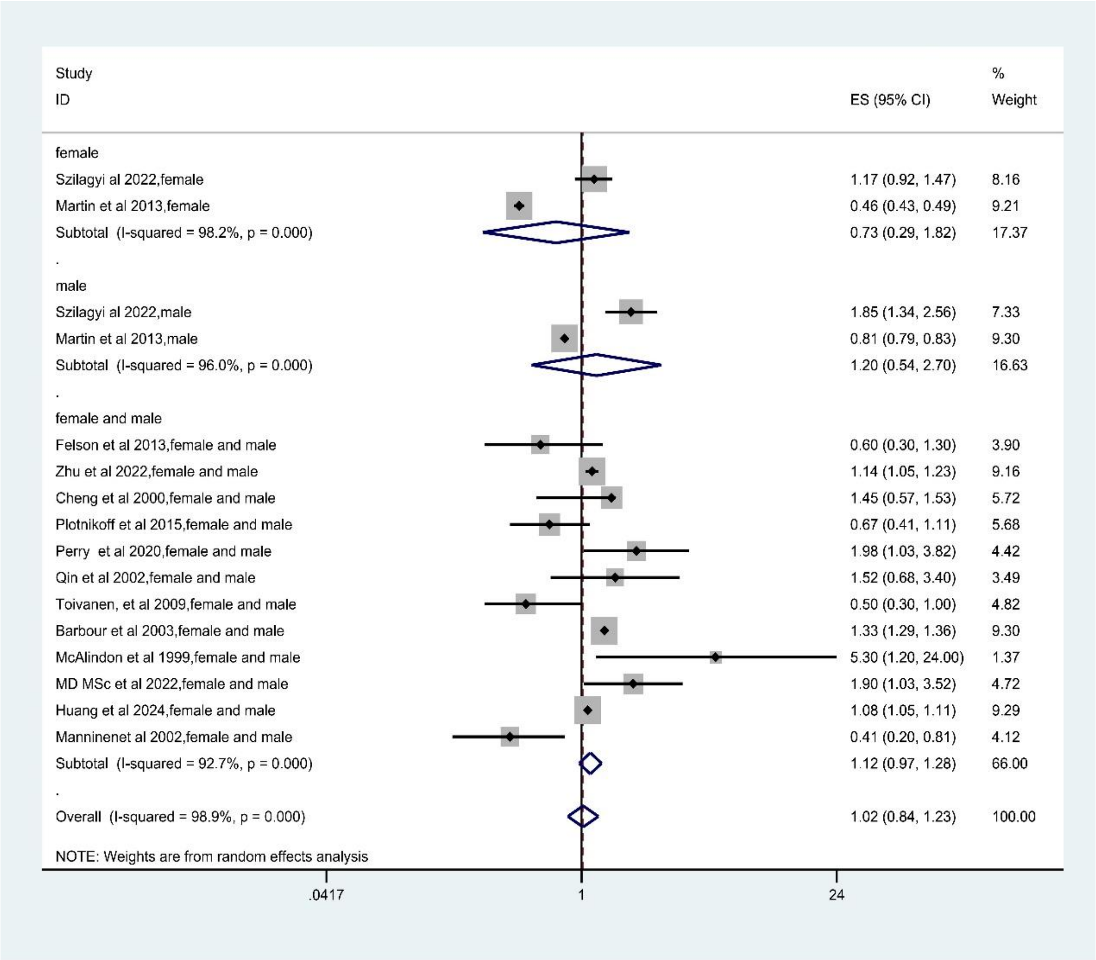

Figure S3. Forest plot of a random effects meta-analysis including 16 risk estimates of knee osteoarthritis for a high versus low level of PA, grouped by sex

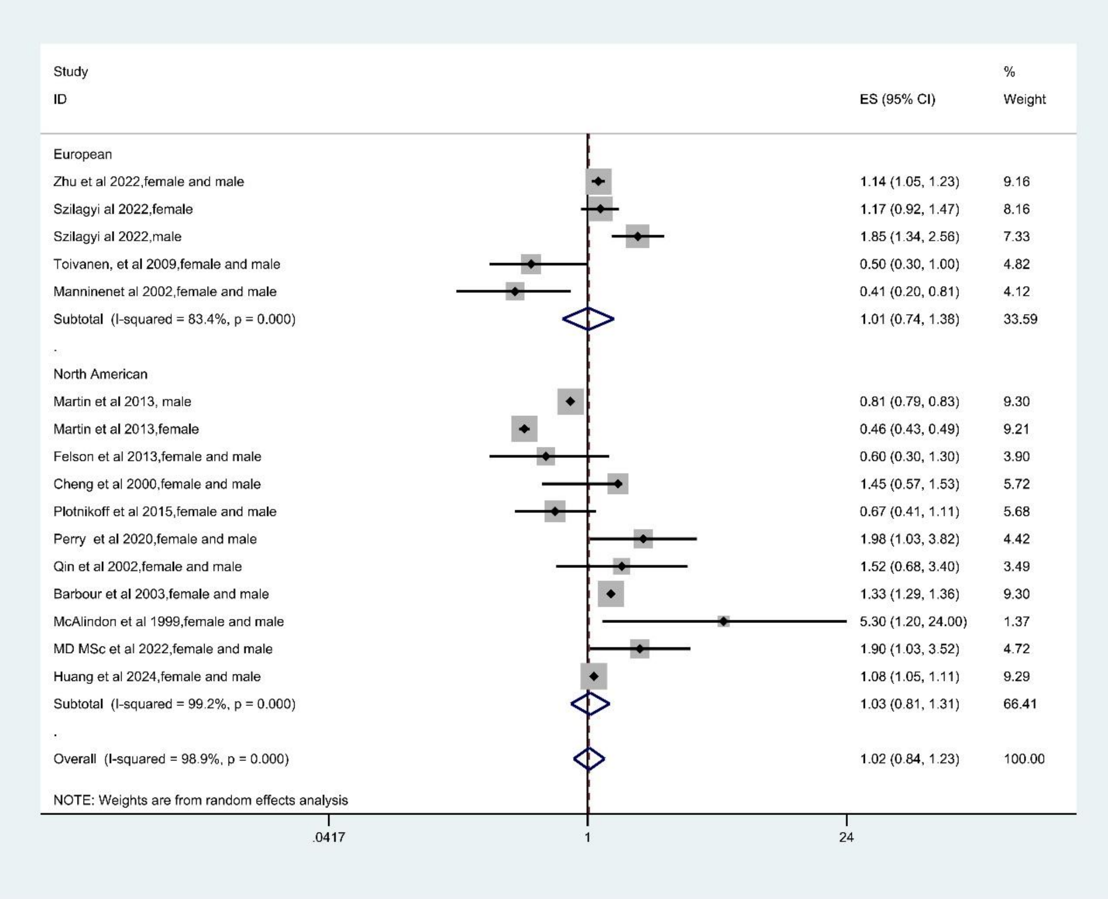

Figure S4. Forest plot of a random effects meta-analysis including 16 risk estimates of knee osteoarthritis for a high versus low level of PA, grouped by region

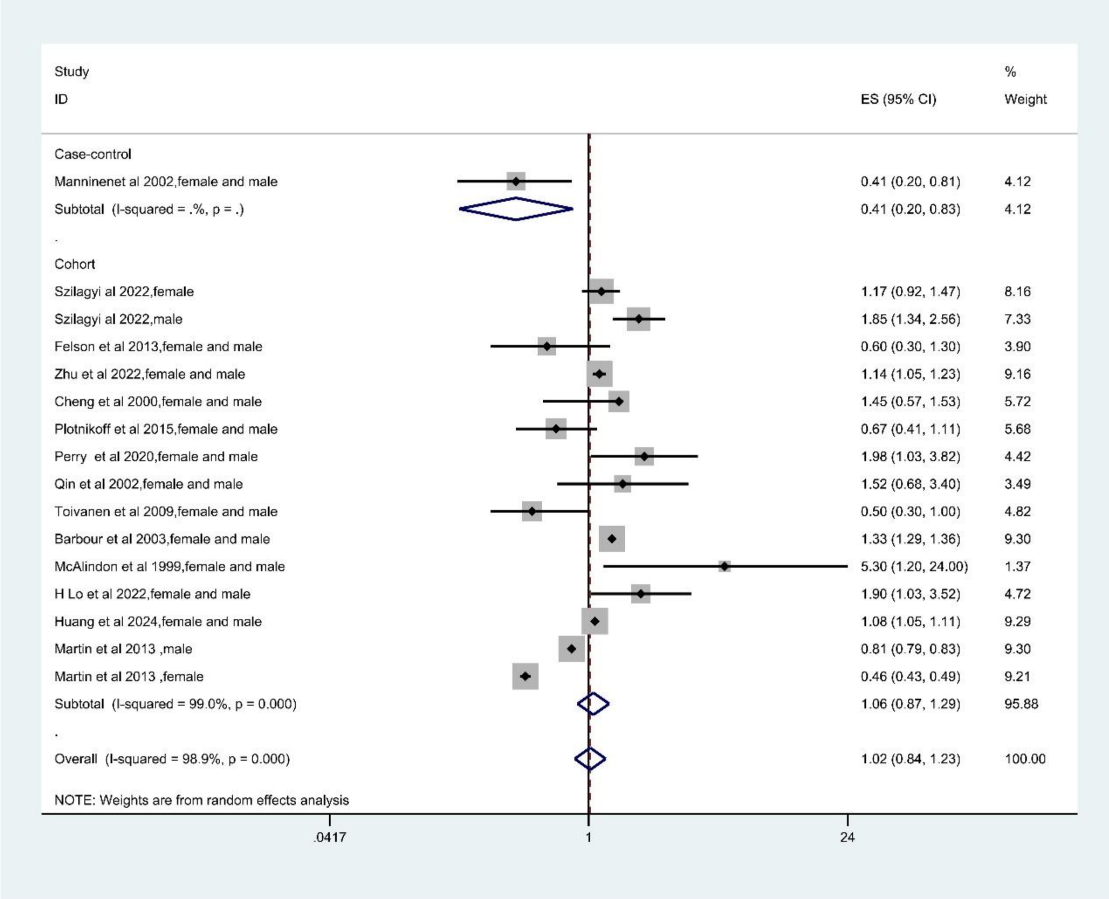

Figure S5. Forest plot of a random effects meta-analysis including 16 risk estimates of KOA for a high versus low level of PA, grouped by type of design

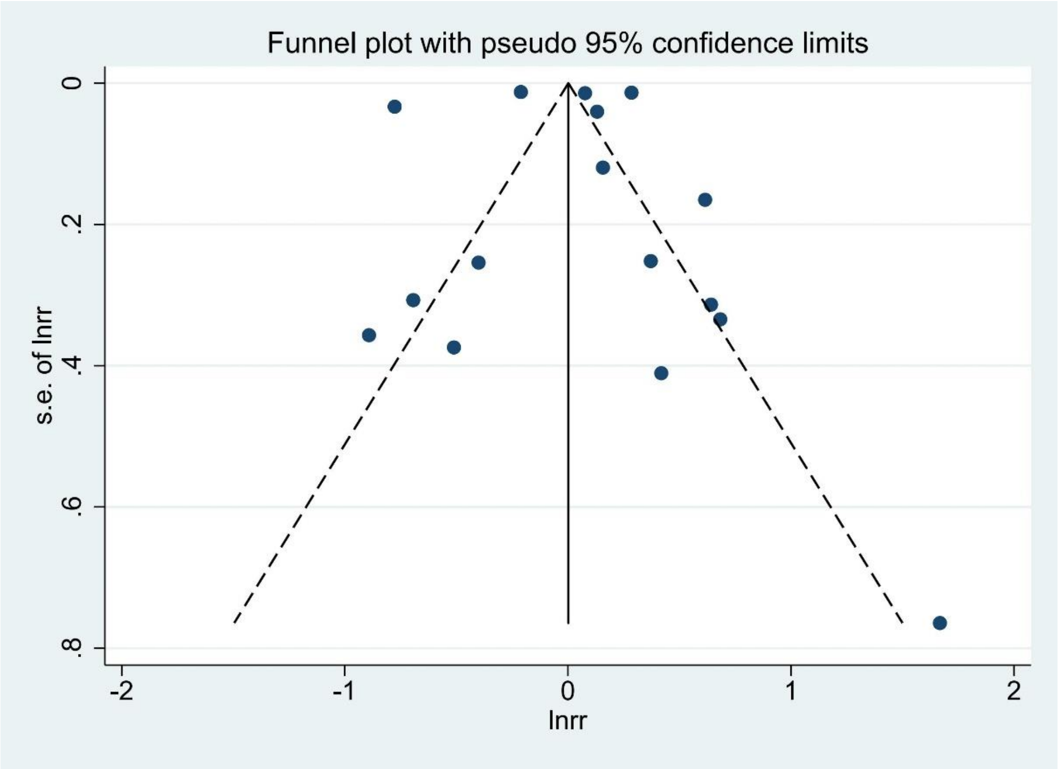

Figure S6. Standardized Funnel plot corresponding to the main random-effects meta-analysis

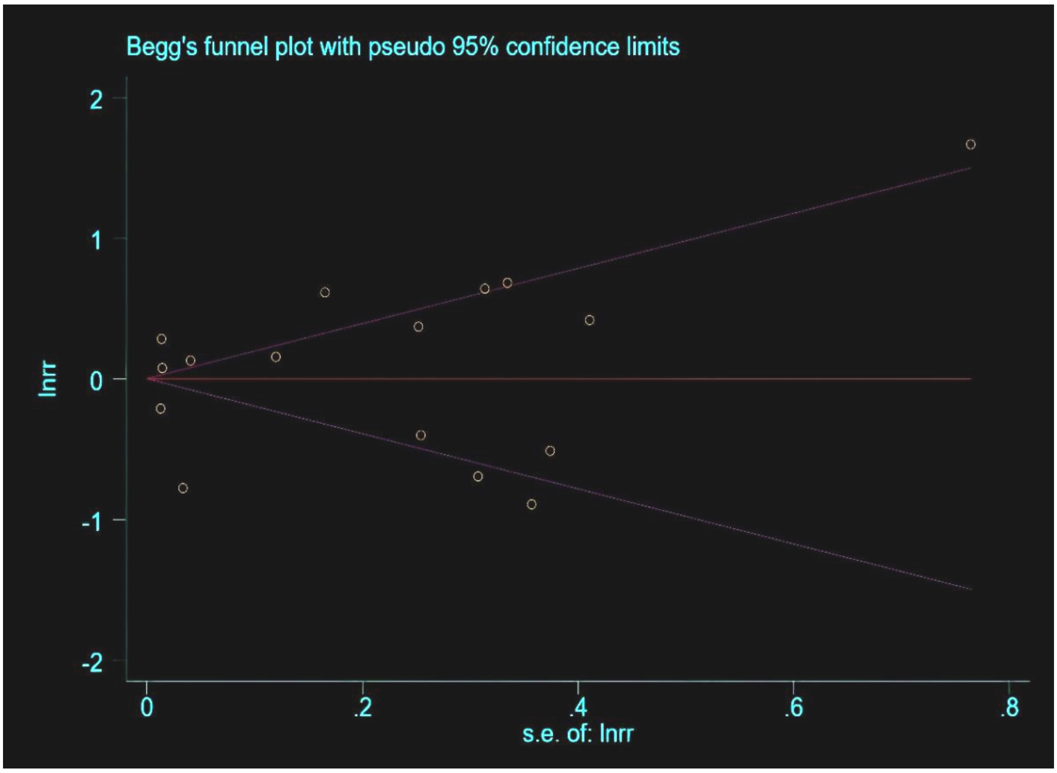

Figure S7. Standardized Beggs rank correlation test corresponding to the main random-effects meta-analysis

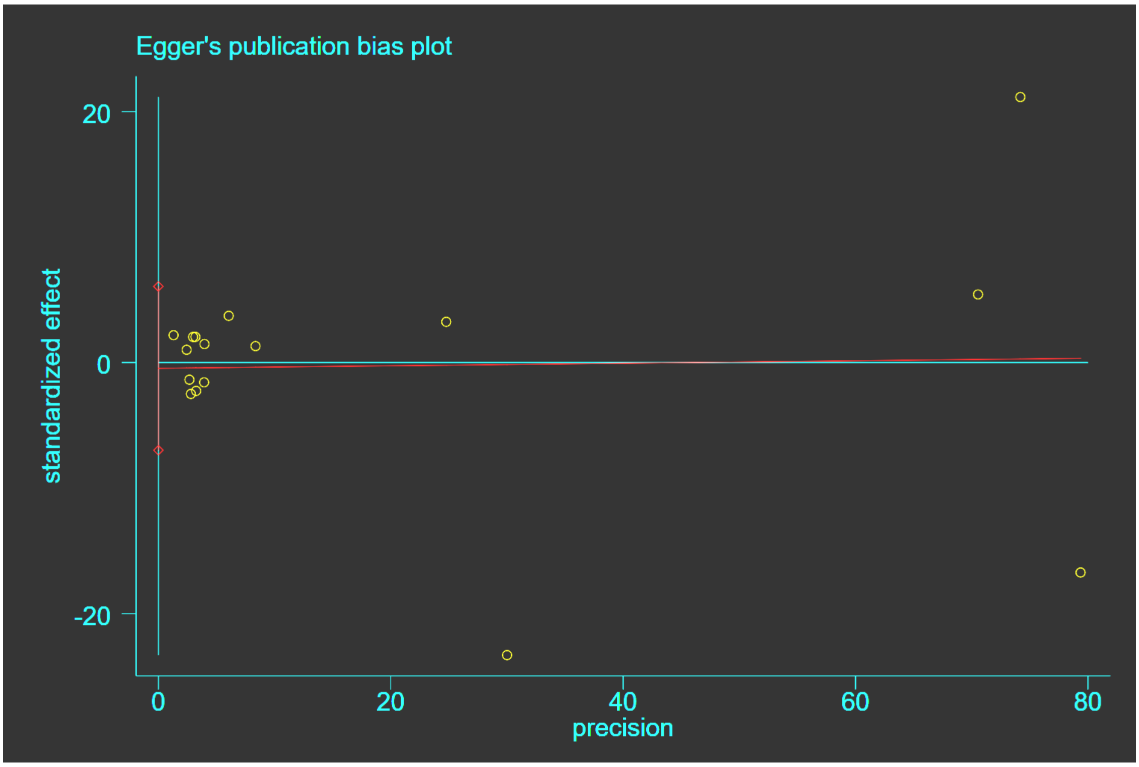

Figure S8. Standardised Egger′s regression test corresponding to the main random-effects meta-analysis

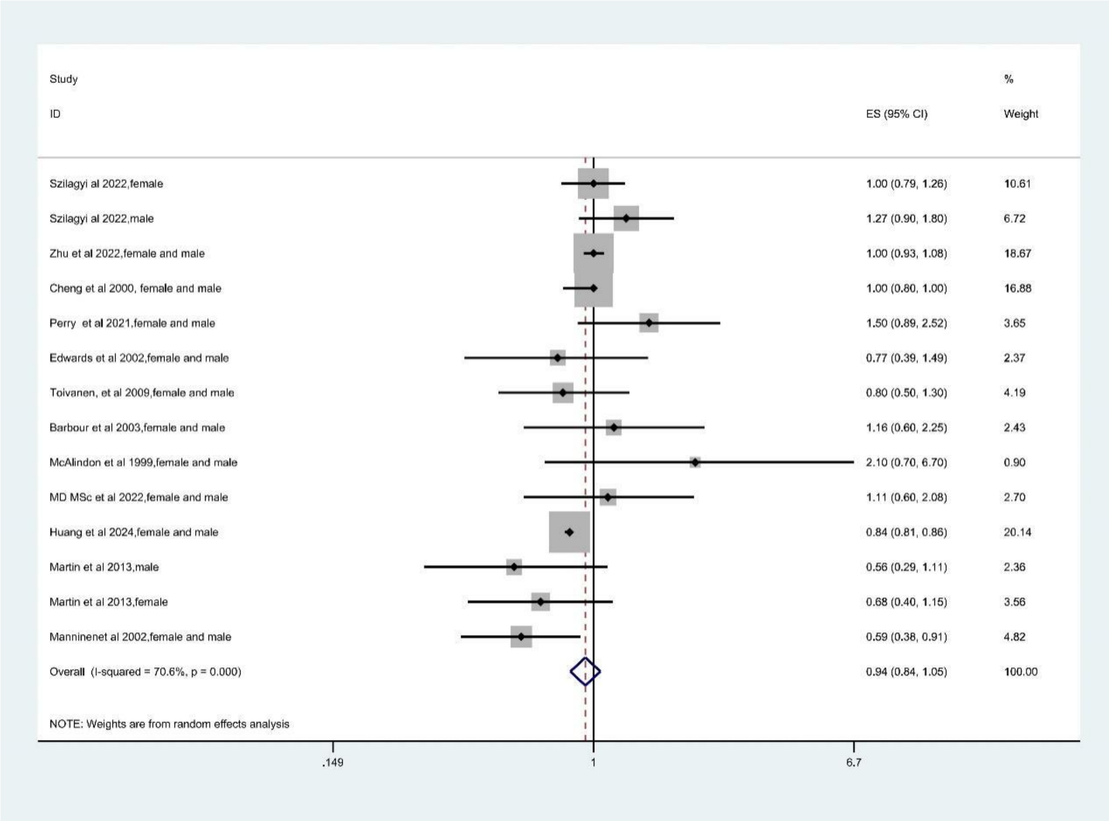

Figure S9. Forest plot of a random effects meta-analysis including 9 risk estimates of knee osteoarthritis for a moderate versus low level of PA

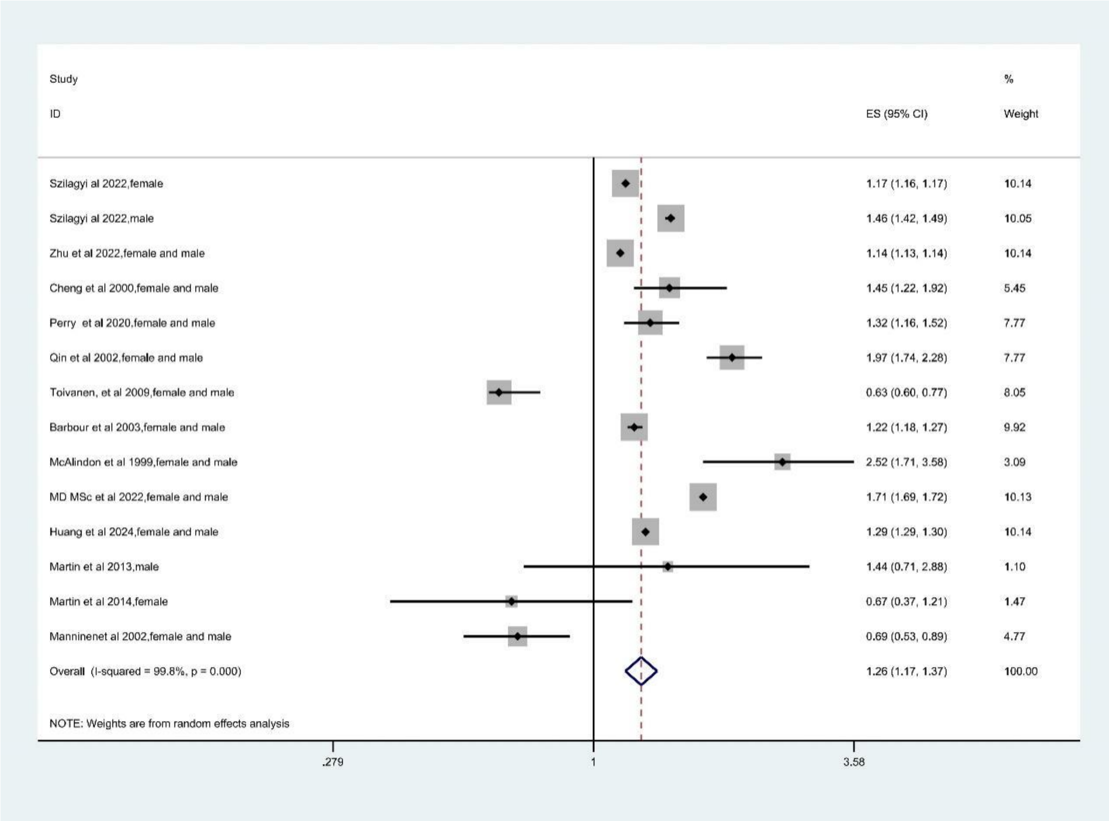

Figure S10. Forest plot of a random effects meta-analysis including 14 risk estimates of knee osteoarthritis for a high versus moderate level of PA

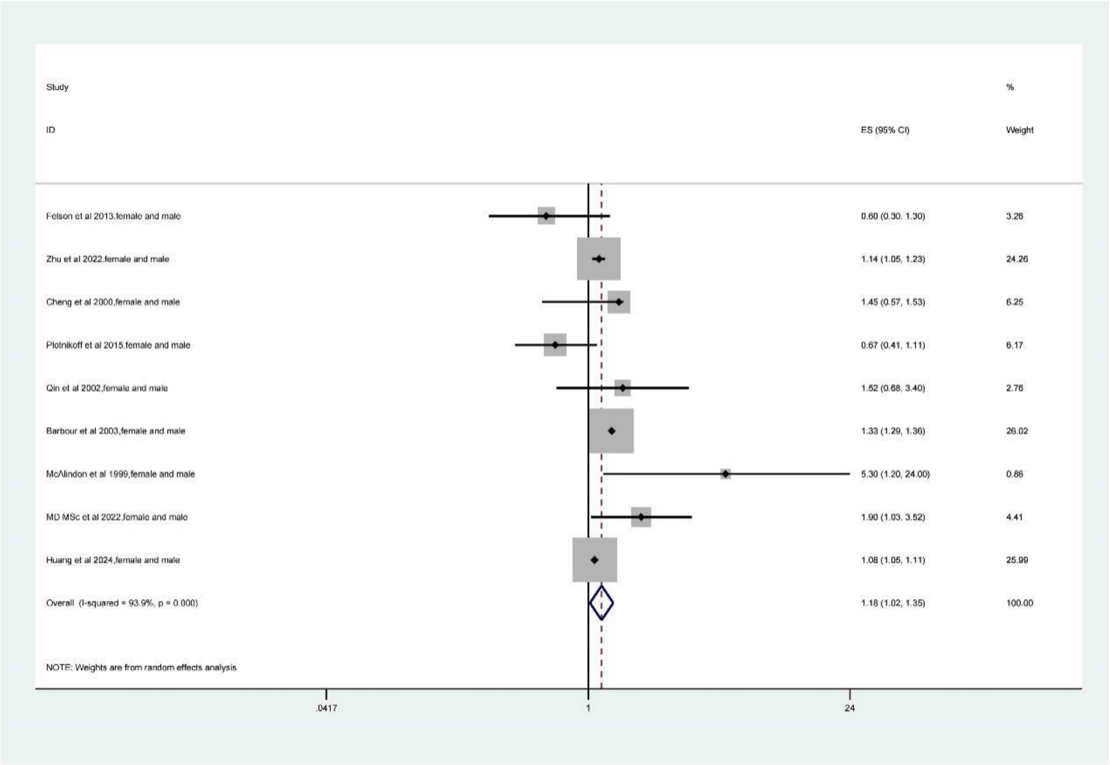

Figure S11. Forest plot of a random effects meta-analysis including 7 risk estimates of KOA for PA guidelines
